# Heterologous expression of *Aspergillus aculeatus* endo-polygalacturonase in *Pichia pastoris* by high cell density fermentation and its application in textile scouring

**DOI:** 10.1186/s12896-017-0334-9

**Published:** 2017-02-16

**Authors:** Dede Abdulrachman, Paweena Thongkred, Kanokarn Kocharin, Monthon Nakpathom, Buppha Somboon, Nootsara Narumol, Verawat Champreda, Lily Eurwilaichitr, Antonius Suwanto, Thidarat Nimchua, Duriya Chantasingh

**Affiliations:** 1 Faculty of Biotechnology, Atmajaya Catholic University, Jl. Jend. Sudirman 51, Jakarta, 12930 Indonesia; 2 Microbial Biotechnology and Biochemicals Research Unit, National Center for Genetic Engineering and Biotechnology, 113 Thailand Science Park, Pahonyothin Rd, Khlong Luang, Patumthani 12120 Thailand; 3 Textile Laboratory, Polymers Research Unit, National Metal and Materials Technology Center, 114 Thailand Science Park, Pahonyothin Rd, Khlong Luang, Patumthani 12120 Thailand

**Keywords:** Acidic pectinase, Bioscouring, Cotton fabric, Heterologous expression, High cell density fermentation

## Abstract

**Background:**

Removal of non-cellulosic impurities from cotton fabric, known as scouring, by conventional alkaline treatment causes environmental problems and reduces physical strength of fabrics. In this study, an endo-polygalacturonase (EndoPG) from *Aspergillus aculeatus* produced in *Pichia pastoris* was evaluated for its efficiency as a bioscouring agent while most current bioscouring process has been performed using crude pectinase preparation.

**Results:**

The recombinant EndoPG exhibited a specific activity of 1892.08 U/mg on citrus pectin under the optimal condition at 50 °C, pH 5.0 with a V_*max*_ and K_*m*_ of 65,451.35 μmol/min/mL and 15.14 mg/mL, respectively. A maximal activity of 2408.70 ± 26.50 U/mL in the culture supernatant was obtained by high cell density batch fermentation, equivalent to a 4.8 times greater yield than that from shake-flask culture. The recombinant enzyme was shown to be suitable for application as a bioscouring agent, in which the wettability of cotton fabric was increased by treatment with enzyme at 300 U/mL scouring solution at 40 °C, pH 5.0 for 1 h. The bio-scoured fabric has comparable wettability to that obtained by conventional chemical scouring, but has higher tensile strength.

**Conclusion:**

The work has demonstrated for the first time functions of *A. aculeatus* EndoPG on bioscouring in eco-textile processing. EndoPG alone was shown to possess effective scouring activity. High expression level and homogeneity could be achieved in bench-scale bioreactor.

**Electronic supplementary material:**

The online version of this article (doi:10.1186/s12896-017-0334-9) contains supplementary material, which is available to authorized users.

## Background

Cotton is an important natural resource used in the textile industry. It is a highly pure natural cellulosic material, comprising approximately 90% cellulose that is organized into strong microfiber structures. Pectin is a major non-cellulosic impurity present in the cuticle and primary cell wall of cotton. It acts as a cementing material for the cellulosic network and as a hydrating agent that controls the movement of water and plant fluids [1], providing firmness and rigidity to the cotton fibers. The backbone of pectin is made of α-1,4-linked D-galacturonic acid residues, in which the carboxylic groups of galacturonic acid are largely esterified with methoxy groups, while the hydroxyl groups on the backbone can be partially acetylated. Xylose, galactose and arabinose are present as side chain sugars of pectin [2]. The hydrophobic nature of pectin is responsible for the non-wetting behavior of the native cotton, which creates difficulty for fabric dyeing in textile processing [3].

Conventionally, pectin and other waxy substances in cotton fabrics are removed by boiling alkali treatment in a process known as scouring. This process makes the fabric more hydrophilic and more accessible for subsequent textile processing. The scouring process carries an environmental burden owing to the large consumption of sodium hydroxide and water (the latter is required for an intensive rinsing step). In addition, scouring also causes non-specific degradation of cellulose, which decreases the tensile strength of fibers, and consequently leads to lower fabric quality [4]. In contrast to the harsh conditions employed in the conventional scouring process, enzymatic processing (bioscouring) can be performed under mild reaction conditions using individual or combinations of different enzymes including lipases, proteases, cellulases and pectinases [1–5]. Among them, pectinases are the most promising [6]. Pectinases release waxes and other non-cellulosic components of the cotton fibers with minimal non-specific damage to the cellulosic structure. Pectinase penetrates the cuticle layer of cotton fiber through cracks or micropores and then partially breaks down pectin in the primary wall matrix [1, 5]. The breakdown of pectin loosens linkages between the cuticle and the cellulose body leading to increased absorbency of the fabric.

According to the mode of action and substrate preference, pectinases are classified into two groups, namely (i) pectin esterases (EC 3.1.1.11) and (ii) pectin depolymerases, which are further divided into polygalacturonases (EC 3.2.1.15, EC 3.2.1.67 and EC 3.2.1.82) and lyases (EC 4.2.2.2 and EC 4.2.2.10) [5]. Polygalacturonases usually work optimally under an acidic range and require no co-factors for their catalysis. Conversely, lyases require divalent metal co-factors and are optimally active under alkaline conditions [7]. Crude pectinase preparations isolated from fungi, for example *Aspergillus* spp. and *Penicillium* spp. that have been tested for potential as bioscouring agents of cotton textiles, possess strong pectate lyase activity. This enzyme works optimally under alkaline conditions, which is compatible with the production of peroxide in the subsequent bleaching step [8, 9] while there has been no report on application of purified or recombinant pectate lyases or polygalacturonases in bio-scouring. In addition to bioscouring, enzymatic starch removal (desizing) using amylases prior to the bioscouring step has been shown to have potential for reducing operating time and energy consumption in textile processing [5, 10]. Combined desizing and bioscouring treatment using amylases and pectinases is hindered by the very different optimal pH of these enzymes. Searching for acidic pectinase working on bioscouring under acidic conditions optimal is thus challenging.

In this study, we aimed to express a recombinant acidic endo-polygalacturonase from *A. aculeatus* in methylotrophic yeast *P. pastoris* using high cell density fermentation. The recombinant enzyme was evaluated in eco-friendly textile processing in comparison to the conventional alkaline scouring process. The developed biscouring process has demonstrated for the first time the action of polygalacturonase on textile scouring with advantages on improved physical properties of the fabrics and allows further optimization of the one-step desizing-scouring for textile processing.

## Methods

### Strains, culturing conditions, genes, and primers

The endo-polygalacturonase gene (*endoPG*), encoding the mature endo-polygalacturonase (EndoPG) without a signal peptide was synthesized based on the coding sequence of *A. aculeatus* ATCC16872. The codon usage was analyzed by Codon Usage Analyzer [11] and optimized for expression in *P. pastoris* using Gene Designer Software [12]. The optimized gene was synthesized by Genescript (Piscataway, NJ, USA). *Escherichia coli* DH5α was used as a host for plasmid propagation. *E. coli* was propagated in low salt Luria-Bertani (LB) medium (1% (*w/v*) tryptone, 0.5% (*w/v*) NaCl, and 0.5% (*w/v*) yeast extract) at 37 °C. *P. pastoris* KM71 (Invitrogen, Carlsbad, CA, USA) was used as a host for EndoPG expression and was cultured in YPD (1% (*w/v*) yeast extract, 2% (*w/v*) peptone, and 2% (*w/v*) glucose). *P. pastoris* transformants were selected on YPD agar containing 100 μg/mL Zeocin (Invitrogen, Carlsbad, CA, USA). pPICZαA vector (Invitrogen) was used as the expression vector. All synthetic oligonucleotides used in this study were purchased from 1st Base (Selangor, Malaysia).

### Construction of expression plasmids

The expression plasmid carrying *endoPG* gene was constructed by amplification of the *endoPG* in pUC57-EndoPG plasmid with a primer pair, EndoPG-F21 (GCATGAATTCGCACCTACAGACATCGAGAAGAGATC; *Eco*RI site underlined) and EndoPG-R1 (TACATCTAGATTAGCAACTGGCACCGGAAG; *Xba*I underlined) for the native mature enzyme. The amplicon was digested with *Eco*RI and *Xba*I and ligated with pPICZαA-digested vector. The recombinant pPICZαA containing *endoPG* (designated as pPIC-PG1), was transformed into *E. coli* DH5α by the heat shock method according to Sambrook and Russel [13]. Transformants were cultivated and selected on LB plates supplemented with 25 μg/mL Zeocin. The DNA sequences of the recombinant plasmids were verified by Sanger sequencing (1st Base, Selangor, Malaysia).

### Heterologous expression of EndoPG in *P. pastoris* KM71

The plasmid pPIC-PG1 was linearized with *Pme*I (Thermo Scientific, Waltham, MA, USA) and then transformed into *P. pastoris* KM71 by electroporation (Gene Pulser, Bio-Rad, Hercules, CA, USA) according to the Easy Select™ *Pichia* Expression Kit instructions (Invitrogen, Carlsbad, CA, USA). Sixty colonies of putative recombinant clones were randomly selected from YPDS plates (1% (*w/v*) yeast extract, 2% (*w/v*) peptone, 2% (*w/v*) glucose, 18.2% (*w/v*) sorbitol, and 1.5% (*w/v*) bacto agar) supplemented with 100 μg/mL Zeocin. Integration of the gene into the *P. pastoris* genome was confirmed by colony PCR using 5′AOX1-F (5′-GACTGGTCCAATTGACAAGC-3′) and 3′AOX1-R (5′-CGAAATGGCATTCTGACATGG-3′) primers according to Easy Select™ *Pichia* Expression Kit instructions (Invitrogen, Carlsbad, CA, USA).

Five mL of YPD medium was inoculated with a single colony of *P. pastoris* transformant and incubated at 30 °C for overnight with rotary shaking at 250 rpm until the OD_600_ reached 5–6. One milliliter of the seed culture was transferred to 25 mL of the buffered glycerol-complex medium (BMGY; 1% (*w/v*) yeast extract, 2% (*w/v*) peptone, 100 mM potassium phosphate buffer pH 6.0, 1.34% (*w/v*) YNB, 0.0004% (*w/v*) biotin, and 1% (*v/v*) glycerol) and the culture was grown under the same condition as described above for 24 h. The cell pellet was then harvested and resuspended in one-fifth of the original culture volume with methanol-minimal medium (BMMY; 1% (*w/v*) yeast extract, 2% (*w/v*) peptone, 100 mM potassium phosphate buffer pH 6.0, 1.34% (*w/v*) YNB, 0.0004% (*w/v*) biotin, and 3.0% (*v/v*) methanol). Absolute methanol was added to a final concentration of 3% (*v/v*) to induce expression of the heterologous gene. The cell-free supernatant was collected at 1, 2, 3, and 4 days after induction for monitoring protein expression by SDS-PAGE [13]. To remove residual culture media and contaminants, the enzyme used for biochemical characterization was further processed by using ultrafiltration (Amicon® Ultra-15, Millipore) with 50 mM potassium phosphate buffer pH 6. Protein concentration was analyzed with Bio-Rad Protein Assay Reagent based on Bradford’s method (Bio-Rad, Hercules, CA, USA) using bovine serum albumin as the standard [14].

### Enzyme activity assay

Pectinase activity was assayed based on hydrolysis of pectin. 0.34 mL assay reactions contained an appropriate enzyme dilution in 0.5% citrus pectin (*w/v*) in 100 mM sodium acetate buffer and incubated at 50 °C for 10 min. The amount of liberated reducing sugars was determined by the dinitrosalicylic acid (DNS) method using D-(+)-galacturonic acid as a standard [15]. The amylase, Avicelase, and CMCase activities were analyzed using 1% soluble starch, 1% microcrystalline cellulose (Avicel®) and 1% carboxymethylcellulose as the substrates, respectively, according to the reaction conditions described above using glucose as the standard. One unit of enzyme activity was defined as the amount of enzyme that catalyzes the formation of 1 μmol of reducing sugar from substrate per min under its optimal conditions. The experiments were done in triplicate.

### Characterization of recombinant EndoPG

The effects of pH on activity of EndoPG was determined at 50 °C for 10 min with a pH range from 2.0 to 9.0, using 100 mM citric acid-sodium citrate (pH 2.0-4.0), 100 mM acetic acid-sodium acetate (pH 4.0-6.0), 100 mM potassium phosphate (pH 6.0-8.0), and 100 mM Tris-Cl (pH 8-9). The optimum temperature was determined within the temperature range of 30–80 °C at pH 5 in 100 mM sodium acetate buffer. The pH stability of the enzyme was studied by incubation of the enzyme at 4 °C in buffers with varying pH for 24 h before determination of the residual pectinase activity under the standard conditions (50 °C, pH 5.0). Thermostability was determined by pre-incubating the enzymes in 100 mM acetate buffer, pH 5.0 at different temperatures (20–60 °C) for varying time intervals from 0.5 to 3 h before analyzing the residual pectinase activity relative to the enzyme activity without pre-incubation denoted as 100%.

The effect of salts (NaCl and KCl), divalent metal ions (CaCl_2_, CuSO_4_, MgCl_2_, MnSO_4_, ZnSO_4_, FeSO_4_, and CoCl_2_) and divalent ion chelator (EDTA) on the enzyme activity was studied by pre-incubating an appropriate dilution of the enzyme in the presence of the compounds at 1 mM at 0 °C for 30 min before determining the pectinase activity under the standard assay conditions. The enzyme activity in the absence of supplemented chemicals was considered as 100%. The data were analyzed by Dunnett’s multiple comparison test compared to the control.

The specificity of EndoPG was assayed on polygalacturonic acid with varying degrees of esterification. The assay reactions contained an appropriate enzyme dilution with 0.5% *w/v* of non-esterified polygalacturonic acid, [6.7%]- or [55–70%]-esterified-pectin from citrus peel (Sigma-Aldrich, USA) in 100 mM sodium acetate buffer, pH 5 and incubated at 50 °C for 10 min. The amount of released reducing sugars was determined using the DNS method as described above. The substrate specificity was expressed as relative percent activity compared with the activity on polygalacturonic acid.

Esterified pectin (6.7%) was used as substrate for the kinetic studies. The *K*_*m*_ and *V*_*max*_ were determined at different substrate concentrations ranging from 0.2 to 20 mg/mL under standard assay conditions (50 °C with an incubation time of 5 min). The kinetics data were calculated and defined by the SigmaPlot 7 (Systat Software, San Jose, CA).

### High cell density fermentation

The recombinant *P. pastoris* expressing EndoPG was cultivated in high cell density fed-batch fermentation in a 5-L bioreactor (B. Braun Sartorius Ltd., Göttingen, Germany) with the method modified from Charoenrat et al. (2013) [16]. Briefly, the primary inoculum was prepared by inoculating a colony of the recombinant *P. pastoris* into a 125 mL flask containing 20 mL of YPD broth and incubated at 30 °C with rotary shaking at 250 rpm for 24 h before subcultivating 20 mL of the inoculum into 180 mL FM22 medium. The FM22 medium contained: KH_2_PO_4,_ 42.9 g/L; CaSO_4,_ 0.93 g/L; K_2_SO_4_, 14.3 g/L; MgSO_4_ · 7H_2_O, 11.7 g/L; (NH_4_)_2_SO_4_, 5.0 g/L; glycerol, 20.0 g/L; histidine, 2.0 g/L and PTM1, 4.35 mL/L. The trace salt (PTM1) contained: CuSO_4_ · 5H_2_O, 6.0 g/L; KI, 0.08 g/L; MnSO_4_ · H_2_O, 3.0 g/L; Na_2_MoO_4_ · 2H_2_O, 0.2 g/L; H_3_BO_3_, 0.02 g/L; ZnCl_2,_ 20.0 g/L; FeCl_3_, 13.7 g/L; CoCl_2_ · 6H_2_O, 0.9 g/L; H_2_SO_4_, 5.0 mL/L; and biotin, 0.2 g/L. The culture was further incubated at 30 °C with rotary shaking at 250 rpm for 48 h. A 5-L bioreactor vessel containing 1.8 L basal salt medium (2.67% (*v/v*) phosphoric acid 85%, 0.093% (*w/v*) CaSO_4_, 1.82% (*w/v* K_2_SO_4_, 1.49% (*w/v*) MgSO_4_.7H_2_O, 0.413% (*w/v*) KOH, 4% (*w/v*) glycerol) was inoculated with 200 mL of the secondary inoculum. The fermentation parameters were controlled at 30 °C, 500–700 rpm agitation, 30% dissolved oxygen (DO), with 1 vvm aeration, and pH 5.0, adjusted using NH_4_OH. The fed-batch cultivation was divided into four stages: glycerol batch stage, glycerol fed-batch stage, methanol induction stage, and production stage as described previously by Jahic et al. [17]. The batch culture was grown until the glycerol was exhausted and the fed-batch mode was applied by constant glycerol feeding at 18.15 mL/h/L of initial fermentation volume for 4 h. The methanol induction feed was started after 3–5 h to avoid repression of AOX promoter [18]. Then, 100% methanol feed was added continuously into the fermentation culture, starting at a rate of 1.00 mL/h/L fermentation volume for the first 2 h and gradually increasing by 10% increments every 1 h to a target rate of 3.00 mL/h/L fermentation volume, which was maintained during the fermentation process. The pectinase activity, dry-cell weight, and protein concentration of the fermenter culture were monitored throughout the cultivation.

### Enzymatic scouring of cotton fabrics

A 211 g/m^2^ enzymatically desized plain-woven 100% cotton fabric (PYI) (Thanapaisal R.O.P, Samutprakarn, Thailand) with a weight of 3.0 g was placed in a 500 mL container with different concentrations of EndoPG (100, 200, 300 and 400 U/mL) at a liquor to fabric ratio of 20:1 in the presence of 0.2% (*w/v*) wetting agent (Hostapal® NIN liq c, Archroma, Singapore). The enzymatic scouring process was carried out in a DaeLim Starlet II DLS-8080 infrared lab dyeing machine at 40 °C for 1 h in 50 mM sodium acetate buffer, pH 5.0 with a heating rate of 2 °C/min and a shaking rate of 30 rpm. The enzyme was inactivated by boiling in distilled water for 10 min twice. The fabric samples were washed twice with distilled water at room temperature and then air-dried. A control treatment was carried out without enzyme addition. Conventional chemical scouring was performed in a 0.2 NaOH solution at 100 °C for 30 min [9]. All experiments were performed in triplicate.

The fabric weight loss was expressed as a percentage loss in weight of fabric after drying at 80 °C in an air-circulated oven water bath with respect to the initial dry weight of the fabric. The samples were weighed after cooling in a desiccator. Water absorbency was evaluated according to the AATCC test method 79-1995 [19]. Tensile strength test was performed according to the ASTM 5034-09 protocol using a bench top material testing machine (H5K-T UTM, Tinius Olsen, PA, USA) with a 75 mm gauge length at an extension rate of 300 mm/min. Three experiments in warp and weft directions were carried out for each sample.

## Results

### Expression of *A. aculeatus* EndoPG

**Fig. 1:**
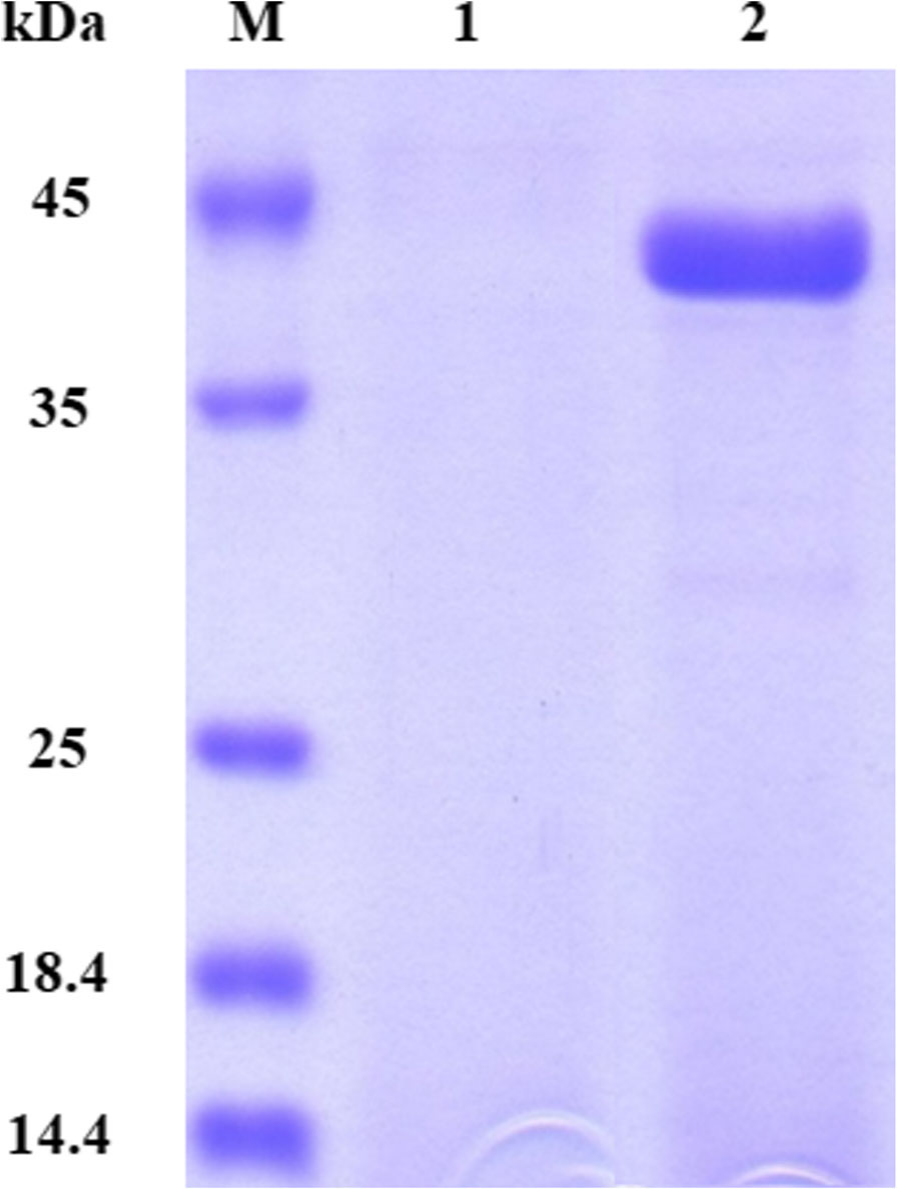
SDS-PAGE analysis of the culture supernatant containing secreted recombinant EndoPG. Lanes *M*: unstained protein molecular weight markers (Thermo Scientific, Illinois, USA). Lane *1* and *2*: culture supernatant of control *P. pastoris* containing pPICZαA and the recombinant clone expressing EndoPG, respectively, after 48 h of methanol induction. The migration of protein band corresponding to the recombinant EndoPG (38.7 kDa) is marked by an *arrow*

In this study, mature *A. aculeatus* EndoPG was expressed in *P. pastoris* KM71 in a secreted form. The *endoPG* gene (1029 bp) was fused in frame to the α–factor secretion signal, under the control of the AOX1 promoter. Induction with methanol led to strong expression of EndoPG in the culture supernatant with high homogeneity (>90%) as shown by SDS-PAGE (Fig. 1). The apparent size of the secreted protein was concordant with the theoretical MW of 38.7 kDa. The highest enzyme expression was obtained after 48 h induction by methanol with the maximal polygalacturonase activity in the shake-flask culture of 503 ± 3 U/mL and a specific activity of 1892 U/mg.

### Biochemical characterization and kinetic study of EndoPG

**Fig. 2:**
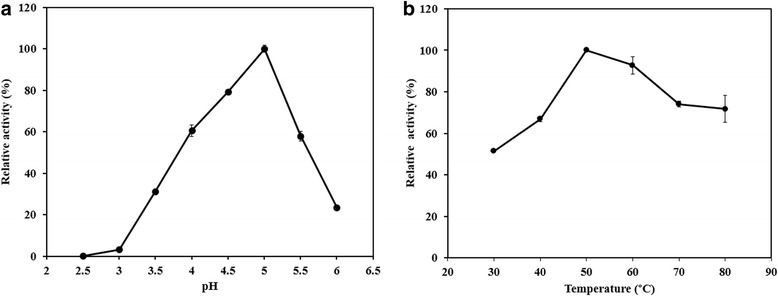
Effects of (**a**) pH and (**b**) temperature on EndoPG activity. The highest activity under the optimal condition (in sodium acetate buffer pH 5 at 50 °C) was considered as 100% relative activity. *Data points* are the mean values and *error bars* represent standard deviations from triplicate experiments

**Fig. 3:**
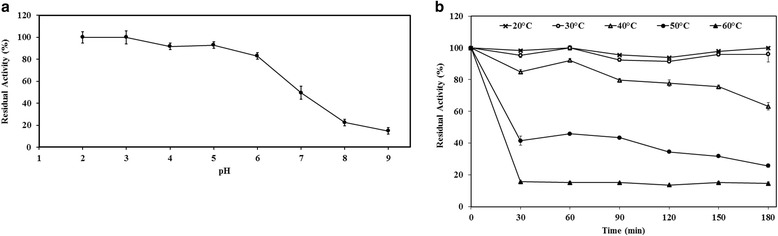
Effects of temperature (part **a**) and pH (part **b**) on stability of EndoPG. Thermostability was determined by incubating the enzyme at different temperatures (20–60 °C) in 100 mM sodium acetate buffer, pH 5 and measuring the residual activity under the optimal enzyme working conditions. For pH stability, the enzyme was incubated at various pH buffers 2.0–9.0 at 50 °C and the residual activity was measured after 24 h. The activity without pre-incubation was denoted as 100%. *Data points* are the mean values and *error bars* represent standard deviations from triplicate experiments

Effects of pH and temperature on the EndoPG activity and stability were studied in order to determine the enzyme’s optimal working conditions (Additional file 1). The recombinant enzyme demonstrated optimal pH and temperature of 5.0 (Fig. 2a) and 50 °C (Fig. 2b), respectively. It retained more than 60% of its initial activity after incubation at 40 °C for 180 min (Fig. 3a). However, the enzyme rapidly lost activity at 50 °C and 60 °C. The pH stability test showed that more than 80% of enzyme activity was retained after incubation in pH 2.0 to 6.0 for 24 h (Fig. 3b). A marked decrease in enzyme activity was observed at pH 7.0 to 9.0.

The effects of metal ions and divalent ion chelator on the activity of EndoPG were examined by determining the enzyme activity in the presence of the additives. Among the various additives tested, Cu^2+^ and Fe^2+^ stimulated the activity of EndoPG (*p* < 0.05) (Table 1). In contrast, Mn^2+^, Na^+^ and Zn^2+^ inhibited the enzyme. K^+^, Li^+^, Mg^2+^ and the divalent ion chelator (EDTA) was found to have no significant effect on the enzyme activity.

**Table 1:** Effects of additives (metal ions and chelator) on the activity of EndoPG

Additive (1 mM final)	Relative activity^a^ (%)
Control	100
Ca^2+^	99.75 ± 2.68
Cu^2+^	112.70 ± 5.02*
Fe^2+^	112.03 ± 0.61*
K^+^	99.90 ± 1.19
Li^+^	100.62 ± 2.74
Mg^2+^	95.55 ± 3.58
Mn^2+^	73.76 ± 2.56*
Na^+^	90.88 ± 2.29*
Zn^2+^	88.81 ± 3.43*
EDTA	98.39 ± 2.73

^a^Mean and standard deviations from triplicate experiments

*Indicates significantly differently from control; *p* < 0.05

The recombinant EndoPG showed the highest activity on polygalacturonic acid, followed by pectin with different degrees of esterification with the relative activity 22–25% of that obtained on the non-esterified substrate (Table 2). There was no non-specific hydrolysis activity against starch or cellulose. The estimated enzyme kinetic parameters of EndoPG for pectin digestion were *K*_*m*_ of 15.14 mg/mL and *V*_*max*_ of 65,451.35 μmol/min/mL.

**Table 2:** Substrate specificity of EndoPG

Substrate	Relative activity^a^ (%)
Polygalacturonic acid	100.00
Pectin (6–7% esterified)	24.99 ± 0.56
Pectin (55–70% esterified)	22.09 ± 0.15
Soluble Starch	nd
Avicel	nd
Carboxymethyl cellulose	nd

^a^Mean and standard deviations from triplicate experiments; *nd* not detected

### High cell-density fermentation

**Fig. 4:**
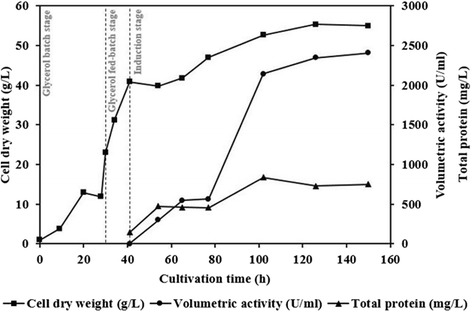
High density fermentation profile of EndoPG production in *P. pastoris*. The cultivation condition during the production stage was maintained at 30 °C and pH 5.0

Production of EndoPG was further studied using high cell-density fermentation approach in a laboratory-scale fermenter. The fed-batch fermentation process comprised three steps including glycerol batch, transition or glycerol fed-batch, and methanol induction or production phases (Fig. 4). The inoculum was prepared in synthetic FM22 medium in order to increase cell density and enzyme production. The glycerol batch stage was carried out for the first 30 h. At the end of glycerol batch stage, the dry cell weight reached 23 g/L. Subsequently, the glycerol fed-batch stage was initiated using constant feeding at the rate of 18.15 mL/h/L of the initial fermentation volume for 4 h. During the glycerol fed-batch stage, the cell dry-weight increased almost two fold from 23 to 41 g_CDW_/L. The EndoPG activity reached the highest level of 2408.00 U/mL in the induction stage (111 h) with the specific production yield of 43,781.81 U/g biomass. The high cell density fermenter thus resulted in 4.8 times greater enzyme production yield compared with the shake-flask culture.

### Bioscouring by EndoPG

The performance of EndoPG on bioscouring of cotton fabric was tested based on weight loss and wettability assay (Table 3). Enzymatic treatment of the fabric with EndoPG led to a marked improvement on water absorbency of the fabric samples compared with the control of no enzyme treatment. The control fabric showed low water absorbency with a wettability time greater than 3 min. Increasing enzyme dosage led to increased fabric wettability (shorter wettability time) and corresponding increased weight loss of fabric. Maximum water absorbency of 16.5 s was observed at the highest enzyme dosage, which was comparable to that measured for fabric scoured by alkali treatment; however, with a lower weight loss of the textile. In addition, the fabrics treated with EndoPG showed a greater tensile strength for both the weft yarn (722.33 N) and warp yarn (986.67 N) compared with that obtained using chemical scouring (Table 4). The results clearly indicated potential and advantages of the bioscouring process using EndoPG as a promising alternative to the conventional alkaline scouring method. Similar results on weight loss, wettability and tensile compared to those obtained using buffer alone were observed using the heat-inactivated enzyme (data not shown).

**Table 3:** Comparison of weight loss and wettability of the fabrics scoured by enzyme (EndoPG) and alkali treatment (sodium hydroxide)

Sample	Weight loss (%)	Wettability (s)
Buffer	0.16	181.20
EndoPG (100 U/mL_scoured solution_)	2.91	19.92
EndoPG (200 U/mL_scoured solution_)	4.22	17.88
EndoPG (300 U/mL_scoured solution_)	5.27	16.50
Sodium hydroxide	7.64	15.84

**Table 4:** Comparison of tensile strength between fabrics scoured by enzyme (EndoPG) and alkali treatment (sodium hydroxide)

Sample	Tensile strength (*N*)	
	Weft Yarn	Warp Yarn
Buffer	741.00	1042.33
EndoPG (300 U/mL_scoured solution_)	722.33	986.67
Sodium hydroxide	713.33	918.67

## Discussion

Heterologous expression of composite enzymes attacking pectin including polygalacturonases, pectate lyases, and pectin esterases from various ascomycetes and basidiomycetes fungi has been reported in yeasts [20–24]. *P. pastoris* is considered an efficient expression system owning to its high production yield of heterologous proteins in secreted forms, allowing cost competitive enzyme production with minimal downstream processing [17, 18]. Recombinant endo-polygalacturonases from *A. niger*, *Bispora sp*., *Phytophthora parasitica* and *Penicillium sp*. produced in *P. pastoris* expression system have been reported with varying biochemical characteristics [20–23]. *A. aculeatus* is a promising producer of multi-component starch and cell wall degrading enzymes with potent application in various biotechnological processes [25, 26]. In this study, an EndoPG from *A. aculeatus* ATCC16872 was expressed as a major protein in the culture supernatant with >90% homogeneity, allowing production of the enzyme with no need for subsequent downstream purification. The enzyme showed a specific activity of 1892 U/mg total protein (50 °C, pH 5) which is comparable to the equivalent recombinant enzyme from *A. niger* [20]. The optimal activity of the enzyme at 50 °C was relatively higher than that of the commercial acid fungal polygalacturonase, which works optimally at 40 °C [9].

Production of EndoPG by high cell density fermentation was achieved yielding maximal activity of 2408 U/mL. The greater yield of enzyme from fermentation compared with batch cultivation is due to the higher cell density achievable in the former, as shown by OD_600_ of 81.60, equivalent to a cell concentration of 40.83 g/L at the exponential glycerol feeding stage before induction by methanol. The benefit of improved yield of enzyme using high cell density fermentation has also been reported for other types of recombinant enzymes expressed in *P. pastoris* e.g., xylanase and cellulase [18, 27]. To our knowledge, our work represents the first report on heterologous expression and high cell density fermentation of polygalacturonase in *P. pastoris* with the highest production yield, 43,781.81 U/g biomass, resulting in the higher enzyme productivity compared to that obtained using batch cultivation using wild type or recombinant microorganisms [22–26].

Bioscouring of cotton fabrics using pectinases has been shown as an interesting approach for reducing chemical and energy consumption in textile processing. Compared to conventional chemical scouring using alkalis such as sodium hydroxide and soaps at high temperature, the bioscouring alternative can result in complete replacement of the scouring chemicals and as the enzymatic process is performed under lower temperature, which leads to substantial saving of cost for energy and waste water treatment [5, 9, 28]. Although EndoPG showed the optimal temperature at 50 °C, the bioscouring process was carried out at 40 °C at which the enzyme showed high catalytic activity with high operational stability (>80% of its maximal activity was retained after 60 min). The lower operating temperature in the scouring step also has advantage on a lower energy cost. Crude pectinases from various bacterial and fungal origins have been used for bioscouring of cottons and other types of textiles e.g., linen and ramie [2]. The use of purified or recombinant composite pectinases on bioscouring has not been previously reported. The recombinant pectinase reported here was shown to be capable of enzymatic bioscouring with a maximal water adsorbancy of 16.5 s, which is in the accepted range for industrial processing (<30 s). However, application of either crude enzymes rich in polygalacturonase or pectate lyase activity alone has been reported to show incomplete scouring on the fabric in some cases [9, 29, 30]. Pectate lyases catalyze trans-elimination of a-1,4-glycosidic linkage in pectic acid and form products with 4,5-unsaturated residues at the non-reducing end. These structural changes increase more polar functional groups and hence the hydrophilicity on the surface of cotton fibers, which could be an important factor for the improved water absorption and dyeability of the treated fabrics. The results thus suggest further formulation of the EndoPG with pectate lyases or other composite pectinolytic enzymes as well as other enzymes e.g., cellulases and cutinases [6, 31] to improve its performance. The EndoPG catalyzed process resulted in fabrics with higher tensile strength compared with the chemical scouring method. This reflected the higher specificity of the enzyme on attacking the polygalacturonic acid backbone of pectin with no decomposition of the cellulose fibers, which is an undesirable side activity of alkali in chemical scouring [9, 28]. This resulted in fabrics with higher strength with lower weight loss from the enzymatic process.

## Conclusion

The applicability of a recombinant cellulase-free polygalacturonase (*A. aculeatus* EndoPG) on bioscouring has been demonstrated. The enzyme can be produced efficiently by high-cell density fermentation in the *P. pastoris* system and showed its potential on completely replacing the use of toxic scouring chemical with substantial energy saving. The EndoPG characterized in this study represents a promising alternative for efficient bioscouring towards establishment of an eco-friendly textile processing industry.

## Additional file

Additional file 1: Effect of pH on EndoPG activity. Effect of temperature on EndoPG activity. Effect of pH on stability of EndoPG activity. Effect of temperature (20–60 °C) on stability of EndoPG activity. RAW data of enzyme activities were calculated and showed as volumetric activity (U/ml) and % relative activity. Standard deviations (SD) were calculated from volumetric activity. (XLS 40 kb)

## Abbreviations

AOX: Alcohol oxidase
CDW: Cell dry weight
EndoPG: Endo-polygalacturonase
